# Electrically stimulated cell migration and its contribution to wound healing

**DOI:** 10.1186/s41038-018-0123-2

**Published:** 2018-07-09

**Authors:** Guangping Tai, Michael Tai, Min Zhao

**Affiliations:** 1 Centre of Advanced Biofabrication, Department of Bioengineering and Environmental Sciences, Hefei University, Hefei City, China; 2 St Catherine’s College, Medical Sciences Division, University of Oxford, Oxford, OX1 3UJ UK; 3 Departments of Dermatology and Ophthalmology, School of Medicine, University of California, Davis, CA 95817 USA

**Keywords:** Wound healing, Ion transport, Endogenous electric fields, Electrical stimulation, Cell migration, Clinical trials

## Abstract

Naturally occurring electric fields are known to be morphogenetic cues and associated with growth and healing throughout mammalian and amphibian animals and the plant kingdom. Electricity in animals was discovered in the eighteenth century. Electric fields activate multiple cellular signaling pathways such as PI3K/PTEN, the membrane channel of KCNJ15/Kir4.2 and intracellular polyamines. These pathways are involved in the sensing of physiological electric fields, directional cell migration (galvanotaxis, also known as electrotaxis), and possibly other cellular responses. Importantly, electric fields provide a dominant and over-riding signal that directs cell migration. Electrical stimulation could be a promising therapeutic method in promoting wound healing and activating regeneration of chronic and non-healing wounds. This review provides an update of the physiological role of electric fields, its cellular and molecular mechanisms, its potential therapeutic value, and questions that still await answers.

## Background

Luigi Galvani provided the first evidence for “animal electricity” in 1794, by demonstrating that muscles contracted when the cut end of a frog sciatic nerve from one leg touched the muscles of the opposite leg [1]. This was subsequently developed by the German physiologist Emil Du-Bois Reymond who made the first recording of endogenous electric currents at a wound [2]. The rapid realization that bioelectricity is highly conserved in animals and plants began to attract broader interest from academics and clinicians alike. Bioelectricity has since been detected at wounds of all animals studied, including humans [3]. Modern techniques such as micro-glass electrodes and vibrating probes or self-referencing electrodes have consistently verified the nature of the endogenous electric fields [3, 4]. Several ionic fluxes were found to be associated with the generation of endogenous electric fields and have also been demonstrated during embryo development, limb regeneration, and wound healing [4, 5]. Disruption of these endogenous electric fields and ionic currents alters normal organ development, tissue regeneration, and wound healing [1–3]. These discoveries not only provide new insights for our understanding of animal electricity, but also point to new directions for therapeutic applications of electric fields in organ regeneration and tissue healing.

## Review

### Active ion transport and endogenous electric fields in wound healing

**Fig. 1:**
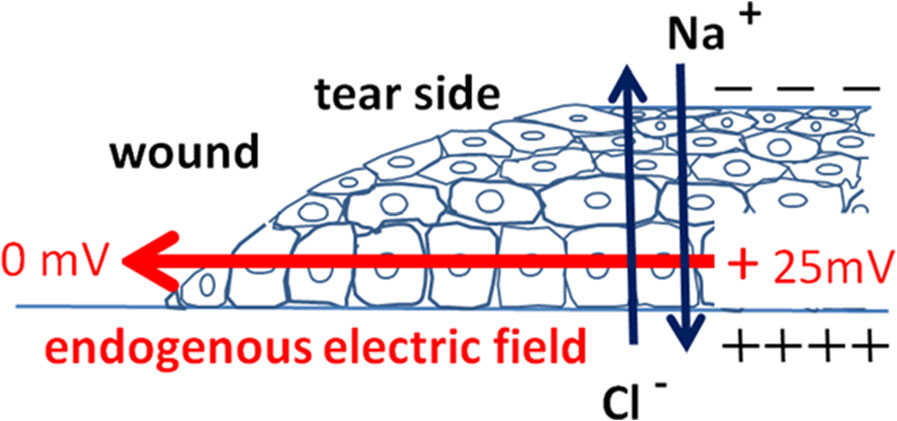
Formation of endogenous electric potential difference due to ions and charged particles flowing out from the wound edge. Skin and other epithelial layers establish laterally orientated endogenous electric fields that point to the wound center upon being wounded. (Adapted with permission from Song et al. [9] Copyright 2002 by National Academy of Sciences)

Skin maintains a surface potential that is negative relative to that underneath the skin, as demonstrated by Foulds and Barker [3]. Undamaged intact human skin maintains an endogenous electric potential and a transcutaneous potential of 20–50 mV. This is generated and sustained by active Na^+^/K^+^ ATPase pumps in the epidermis. Upon injury, ion leakage occurs across wounded cells or cell layers. This establishes a voltage gradient laterally orientated at wounds, pointing to the wound center. The asymmetric ionic flows of mobile charged ions generate the endogenous electric potential. Na^+^, Cl^−^, K^+^, and Ca^2+^ are the main components of the endogenous electric currents (Fig. 1) [4, 5].

Incisional wounds on skin or corneal models are commonly used for measurements of endogenous wound electric fields—for its simplicity and accessibility. In corneal wounds, through the use of a self-referencing electrode, endogenous wound electric fields have been measured: varying from 10 to 60 mV. These fields have an outward current of 4 μA/cm^2^, which immediately appears at the edge of the wound, increases to 10 μA/cm^2^ and persists at the level of 4 to 8 μA/cm^2^. The largest outward currents are found at the wound edge, 0.9 mm from the wound center, in a circular corneal wound model of a diameter of 1.8 mm [4, 5]. Although the wound healing process of incisional wounds on skin or on corneal models are similar to skin burn wounds in patients, the latter is likely to be complicated by dead tissues at the wounds, contamination, and infection [6–8]. There is no report on endogenous electric field measurements on burnt skin due to the technical difficulty, the complex clinical setting, and irregular wound boundaries on both animal models and in patients with burns.

### The electric field provides cues for directed cell migration

The existence of hitherto under-appreciated endogenous electric currents led to extensive exploration and study. The effect of external electrical stimulation has been widely tested on different types of cells in vitro*.* Multiple types of cells show directional migration in an external electric field, a phenomenon termed electrotaxis or galvanotaxis. Human epithelial cells from the skin or the cornea [6, 7], fibroblasts [9], lymphocytes [8], macrophages [10], endothelial cells [11], and neuronal cells [12] are all responsive to small applied electric fields. Applied electric fields have been demonstrated to affect cell migration, proliferation, and orientation of cell division in both individual cells and cell sheets [13].

Keratinocytes, corneal epithelial cells, and osteoblasts are responsive to voltages as low as approximately 10–25 mV/mm (less than 0.5 mV across a cell of 20 μm in diameter), which is within the physiological range [6, 14–16]. Interestingly, epithelial cells migrate directionally toward the cathode in an electric field [6, 9]. Stromal fibroblasts from the cornea and osteoclasts migrate toward the anode [16, 17]. Cell migration in an electric field was initially oversimplified and suggested to be due to the passive movements of charged particles. The passive movement hypothesis is now considered incomplete, as the electrotaxis direction does not always coincide with the direction of movement of charged macromolecules within cells [18]. During electrotaxis, cells extend membrane protrusions and relocate membrane receptor proteins actively. Charged macromolecule receptors on the cell membrane such as the Con A receptor and epithelial growth factor receptors (EGFR) are found in an asymmetric distribution after electric field treatment [18, 19].

### Electric fields elicit a stem cell regenerative response

The effect of electric fields on cells in wounds extends beyond cell migration cues. Evidence of electric field-elicited stem cell regenerative responses are emerging. Electric fields affect stem cell differentiation, and electric fields are capable of powering stem cell regenerative potential. However, the biophysical mechanisms by which stem cells sense, interpret, and transform electrical cues into biochemical and biological signals remain unclear.

Simple direct current pulsed treatment could control the fate of neural stem and progenitor cells (NPCs). Chang et al. [20] demonstrated that square wave direct current pulses (magnitude 300 mV/mm at a frequency of 100 Hz) induced morphologic and phenotypic changes in mouse neural stem and progenitor cells, in stem cell maintenance medium. The NPCs were induced to differentiate into neurons, astrocytes, and oligodendrocytes. The length of primary processes and the amount of branching significantly increased after stimulation by direct current pulses for 48 h.

Adipose-derived stromal cells play a key role in skin wound regeneration. Hammerick et al. [21] reported murine adipose-derived stromal cells (mASCs) migrated toward the cathode in direct current fields of physiologic strength and exhibit dose-dependent migration. Electric fields also caused stromal cells to orient perpendicularly to the field vector, and electric fields elicited a transient increase in cytosolic calcium.

Furthermore, Llucià-Valldeperas et al. [22] reported that the use of electric field-trained adipose tissue-derived progenitor is advisable for tissue regeneration (cardiac). The electric field-stimulated cells became better aligned to patterned surfaces, making cells more suitable for cardiac regeneration, compared to controls; electrically stimulated cells showed phenotypic changes: better alignment and perpendicular reorientation to the electric field. This was effective in cell suspension or within engineered tissue.

Mesenchymal stem cells (MSCs) have been well established to play a key role in tissue regeneration and wound healing. Zimolag et al. [23] found that the reaction of MSCs to electric field stimulation was very rapid, occurring within 1 min. Mesenchymal stem cells migrate toward the cathode, and interruption of PI3K and Arp2/3 had the most pronounced effect on electrotaxis in MSCs. On the other hand, macrophages, the other key player in wound healing, migrate toward the anode. However, macrophage electrotaxis is mostly dependent on Rho family of small GTPases.

Factors released by bone marrow (BM)-MSCs recruit macrophages and endothelial lineage cells into the wound, thus enhancing wound healing. BM-MSCs secrete distinctively different cytokines and chemokines: BM-MSC-conditioned medium significantly enhanced the migration of macrophages, keratinocytes, and endothelial cells and the proliferation of keratinocytes and endothelial cells. Electric field stimulation might affect stem cell fate change and functional activation [20]; electric field stimulation may provide a simple yet effective approach in promoting tissue repair and tissue regeneration.

### Revealing intracellular signaling induced by electrical stimulation

It is conceivable that electrical stimulation can execute its physiological functions or even overriding physiological signals through integration with existing intracellular regulatory mechanisms. Although details are still unfolding, discovery of the asymmetric distribution of membrane receptors to cathodal or anodal facing sides was pioneered by Poo et al. [18] who showed Con A receptor asymmetries after direct current electric field application. It has been reported that the EGFR were redistributed asymmetrically after application of a direct current electric field, on both keratinocytes and corneal epithelial cells [6, 19]. This notion is further supported by recent evidence of the asymmetric distribution of activated downstream intracellular molecules of signaling cascades such as increased lamellipodial Ca^2^^+^ sparks, relocation of extracellular signal-regulated kinase 1, 2 (ERK1, 2), and pERK1, 2 (phosphorated ERK1, 2). [24, 25]

**Fig. 2:**
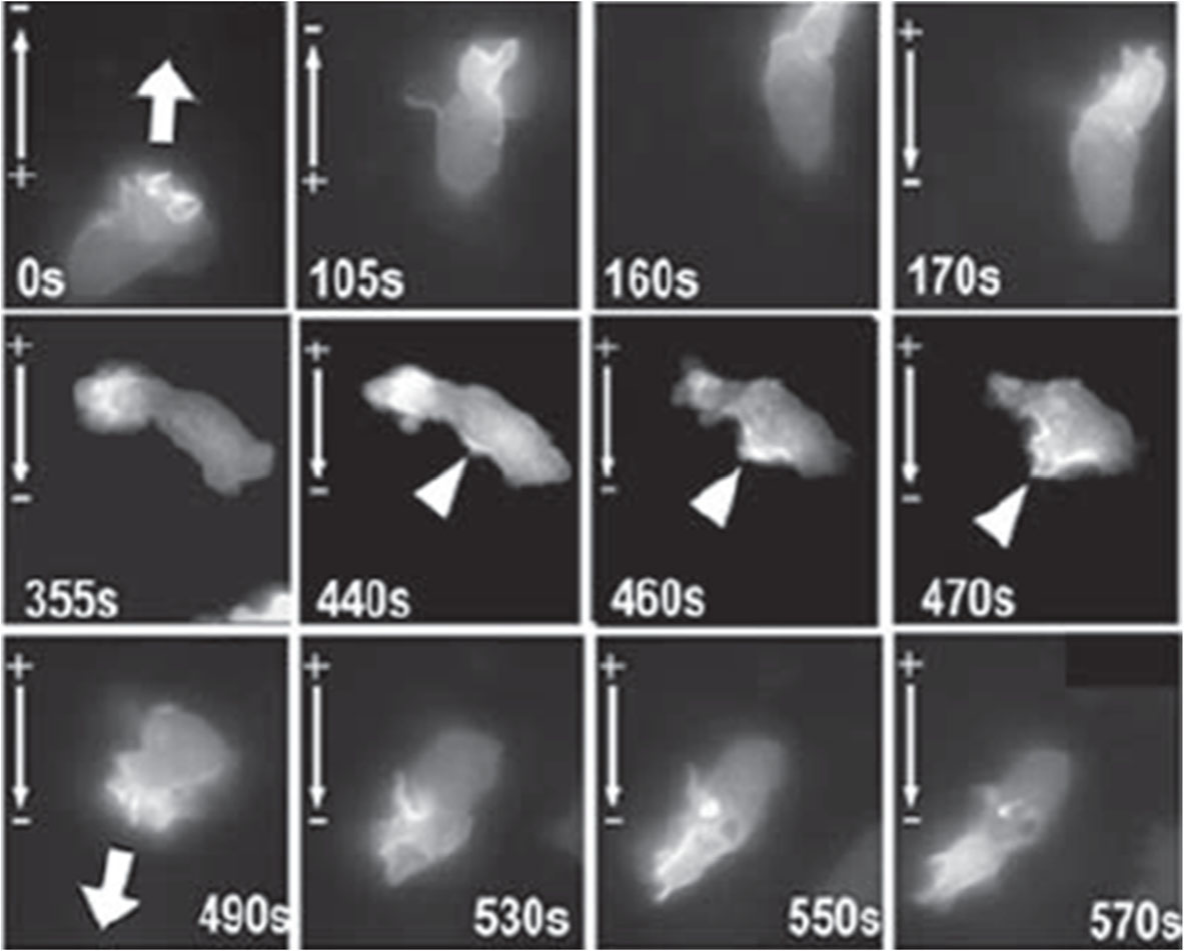
An applied electric field induces polarized activation of PI3K kinase. AKT PH domain-GFP fusion protein reports activation and polarization of PI3K kinase/AKT pathway in an HL-60 differentiated neutrophil-like cell in an electric field. The activation is at the leading edge, when the field polarity is reversed (at 170 s), the new activation site and leading edge form (at 440 s), and the cell moves in the opposite direction. The figure is reproduced with permission from the article of Zhao et al. [26] (Copyright 2006 by Springer Nature)

Using pharmacological and genetic approaches, two key signaling molecules were discovered to be required for electric field-induced migration [26]. Electric field activates the PI3K-AKT (phosphoinositide-3 kinase-AKT serine/threonine kinase) pathway, producing PIP3 (phosphatidylinositol-3,4,5-bisphosphate) and activation of AKT (Fig. 2), which induces asymmetric intracellular signaling cascades. AKT activation is critical for cellular responses following wounding, such as cell migration, survival, and proliferation. Genetic disruption of PI3K (p110), the catalytic subunit of PI3 kinase γ, abolishes directed movements in epithelial healing. In contrast, deletion of the Pten (phosphatase and tensin homolog) gene, an antagonist of the PI3K-AKT pathway, enhances the PI3K-AKT signaling axis and enhances the electric field-induced cellular responses [26]. Through the use of green fluorescent proteins, the dynamics of PI3K activation induced by an applied electric field was visualized by AKT (Pleckstrin homology domain-green fluorescent protein) PH domain-GFP fusion protein in real time, as shown in Fig. 2. Pleckstrin homology domain (PH domain) binds PtdIns (3,4)-P2 phosphoinositides; it marks the activation of PI3K and distribution of phosphoinositides. This demonstrates there are molecules which interact with external electric fields to direct cell migration.

Recently, in search of cellular targets that respond to galvanotaxis, Nakajima et al. [27] screened a library of 381 genes encoding ion channels, pumps, and transporters using a siRNA knockdown strategy on a human corneal epithelial cell line (hTCEpi cells). The screening identified 35 genes that showed significant effects on galvanotaxis. In particular, knockdown of KCNJ15, a gene encoding the K^+^ channel Kir4.2, completely abolished galvanotaxis while maintaining the normal migration speed, which was further verified on the HaCaT cell model, a spontaneously immortalized human keratinocyte line which migrates to the cathode, and on the MDA-MB-231 cell model, a human breast adenocarcinoma line which migrates to the anode. Genetic and pharmacological evidence revealed an interesting two-molecule model of K^+^ channel/Kir4.2, and intracellular polyamines were proposed to be instrumental in external electric field polarity sensing. Initially, a weak extracellular electric field redistributes positively charged polyamines, which then bind to K^+^ channel Kir4.2 and consequently regulate the flux of potassium ions. K^+^ channel Kir4.2 activity alteration induced local changes in membrane potential, osmolality, and the ionic environment. It consequently interacts with the previously established intracellular PI3K/AKT pathways and ultimately affects actin polymerization, membrane protrusion, and cell migration. Nevertheless, this new two-molecule model hypothesis is not an exhaustive account; other mechanisms such as the potassium transporter Trk1p, sodium channel ENaC, calcium channels, and integrin molecules have also been shown to be required for electric field sensing in yeast and in keratinocytes [28]. Electrical stimulation is likely to affect mammalian cells and yeast through the same or similar mechanisms, yet these responses are likely to be cell-type specific and stimulation parameter-dependent. For example, there are reciprocal interactions between lipid rafts and integrin, which are necessary for direct current-induced polarization of intracellular signaling molecules, while in AC fields, polarization is frequency-dependent [29]. Microenvironmental factors such as hypoxic preconditioning can accelerate electric field-guided directional migration of keratinocytes; this effect was both oxygen tension- and preconditioning time-dependent [30]. Furthermore, in Dictyostelium, PI3Ks and cGMP mediate cathode-directed signaling and migration can be switched between cathode-directed and anode-directed signaling through intracellular cGMP levels [31]. The roles of other known chemotactic signals in electrotaxis remain unclear.

### Electrical stimulation for treatment of chronic and non-healing wounds

Electrical stimulation may work on each stage of wound healing including decreasing inflammatory cell infiltration by macrophages and leukocytes [32] and providing directional cues in the homing of stem cells to wounds [33]. The healing effects of external electrical stimulation appear to be conserved in wound healing models across different species [26]. The power of electrical stimulation to accelerate healing has yet to be fully explored.

There are accumulated data of electrical stimulation activating wound healing in a clinical setting. Thakral et al. [34] reviewed a total of 21 randomized clinical trials in databases that used electrical stimulation for wound healing, excluding five studies with less than eight subjects. Electrical stimulation was associated with faster wound area reduction in 14 out of 16 clinical trials, regardless of differences in electrical waveform and duration of electrical stimulation. Electrical stimulation is an effective adjunctive therapy and is currently underutilized.

Lala et al. [35] reviewed a total of 599 articles from which only 15 clinical studies were selected for inclusion in the meta-analysis. Five studies demonstrated that electrical stimulation decreased ulcer size significantly by 1.32%/day, with a 95% confidence interval (CI) of 0.58–2.05. Electrical stimulation accelerated pressure ulcer closure significantly (*P* < 0.001) compared to standard wound care in patients with spinal cord injury. This is consistent with the review of clinical studies by Smit et al. [36] on the effectiveness of electrical stimulation for patients with spinal cord injury.

Khouri et al. [37] also analyzed 29 randomized clinical trials with 1510 patients and 1753 ulcers. Khouri et al. concluded that the overall efficacy of electrical stimulation on would healing was significant (with a standard mean difference 0.72 and 95% CI of 0.48–1). In particular, electrical stimulation was more effective on pressure ulcers compared to venous and diabetic ulcers. The efficacy tended to be inversely associated with the wound size and duration. Furthermore, Khouri pointed out that unidirectional high-voltage pulsed current (HVPC) with the active electrode over the wound was the best evidence-based protocol to improve wound healing.

Ud-Din and Bayat [38] evaluated a total of 48 studies with different modalities including direct current, alternating current, HVPC, low-intensity direct current, and electrobiofeedback electrical stimulation. All electrical stimulation modalities demonstrated positive effects on cutaneous wound healing with different causes. However, no single modality was advocated as the most optimal for the treatment of cutaneous wound healing.

Recently, Ashrafi et al. [39] systematically reviewed 11 clinical studies with a total of 490 subjects. It was confirmed that electrical stimulation results in significantly faster healing rates compared with controls. This is further supported by Ramadan et al. [40] who confirmed that electrical stimulation treatment showed a 144% increase in healing rate over control wounds. However, the optimal stimulus parameters and treatment schedule of electrical stimulation for chronic wounds remain inconclusive; research designs remain a common problem among most reported clinical case studies. More detailed analysis reviewed by Houghton [41] concluded that not all forms of electrical stimulation produce beneficial results; only studies using certain electrical stimulation protocols such as monophasic pulsed current applied to the wound and biphasic pulsed current that is applied for 2 h daily to periulcer skin at intensities which produce motor responses have consistently demonstrated positive results.

Despite the fact that the clinical research designs were not comparable in most studies, Kloth [42] reviewed six clinical studies and found that electrical energy is a useful indicator and the total charge dosage range of 250–500 ų Coulomb/s represents a small window of electrical energy that has been shown to produce favorable wound healing results. The same electrical energy parameters used in different devices and in different studies yielded unexpected reproducible wound healing results in four out of six studies.

The reported studies and clinical pictures are complex, but the results of electrical stimulation are encouraging: the majority of clinical trials showed significant improvement in wound area reduction or wound healing compared to standard care. The electrical stimulation treatment is relatively safe, effective, and well tolerated. However, rigorous clinical trials are needed to aid understanding of optimal dosing, timing, and type of electrical stimulation to be used.

### Electrical stimulation devices for wound healing

The emerging evidence of beneficial effects of electrical stimulation on wound healing seems undeniable though inconclusive in some cases. The demand from clinicians for wound healing is increasing, especially for chronic and non-healing wounds; this has stimulated the development of novel treatment devices. Among other available treatment modalities, including hyperbaric oxygen therapy and negative pressure wound therapy, electrical stimulation is an attractive option [43].

The principle of externally applied electric currents in wound tissue is intended to mimic the endogenous currents which facilitate wound healing. Medical devices are designed to conduct via traditional electrodes, arrays of batteries, or electric currents induced in tissue by pulsed electromagnetic waves [44]. There are excellent reviews on electrical stimulation technologies and electrical stimulation devices for wound healing and antibacterial purposes by Kloth and Anderson et al. [42, 43].

The increasingly diversified electrical stimulation devices can be broadly separated into two categories: direct current and pulsed current (PC). The most common type is the low-voltage monophasic PC and the twin-peaked waveform of high-voltage monophasic PC. HVPC typically has a very short duration (20–60 μs). Their application and clinical studies on wound healing has been reviewed [39–41]. In this review, only three newly added electrical stimulation devices are discussed.

POSiFECT® wound dressing product is a disposable bioelectric device [42, 45]. It contains a miniature electric circuit that delivers a direct current microamperage current to the wound. The anode is a flexible metal ring embedded into hydrogel in the dressing, and the cathode is a dime-sized electrode embedded in hydrogel that is applied directly to the wound bed. The current comes from two built-in lithium batteries. Clegg and Guest [46] showed that POSiFECT® RD bioelectric wound dressing is effective for activating chronic wound healing.

Another electric dressing product is Procellera™. The dry dressing Procellera™ is electrically inactive, and its batteries are claimed to be activated when moistened with wound exudate or by saline. The manufacturer claims that it is able to generate a sustained voltage (2–10 mV) for up to 7 days on the wound surface [45, 47].

However, applying electrical stimulation to wounds remains a technical challenge as direct contact of the electrode with the wound bed causes pain and risks infection. To overcome this, developing a device that allows delivery of electrical stimulation without direct skin contact would be ideal. Wirsing et al. [48] reported a new non-invasive, electrical stimulation device, which transfers a 1.5-μA current to any surface wound from a distance, using oxygen’s and nitrogen’s ability to exchange electrons. After treatment twice or three times per week for 45–60 min per session, the wireless micro-current stimulation treatment significantly accelerated wound healing for patients with chronic wounds of different etiologies. It has proved to be an easy-to-use, non-invasive, time-efficient treatment.

A major caveat is that, in most clinical study settings, no device has been approved by the FDA for promotion of wound healing. While electrical stimulation devices such as Procellera™ has been used on patients with partial-thickness wounds, full-thickness wounds, pressure ulcers, venous ulcers, and diabetic ulcers in studies, the FDA has only approved Procellera™ for anti-infection use.

### Limitations in the utilization of electrical stimulation

Recent systematic reviews provide positive recommendation regarding the effectiveness of electrical stimulation to increase wound healing [34–39, 42–44]. However, most devices are electrode-based and need skin contact to establish the electric circuit. Application on the wound risks infection and skin irritation. Minimizing the interruption to the wound bed and the wound healing process remains technically challenging.

Electrical stimulation parameters for specific wound condition (diabetic chronic wounds, pressure sore wounds, and simple wounds) remain to be optimized. A common issue in reported electrical stimulation investigations is that of limited sample sizes. Industry-sponsored phase III clinical trials can remedy this. In addition, many case studies looked at percentage change in wound area as the primary outcome rather than complete delayed wound healing, which normally happens at 12 to 20 weeks.

Electrical stimulation duration remains controversial. Under experimental settings, in vitro endothelial cell orientation was seen as early as 4 h after the application of an electric field with 100 mV/mm. In clinical settings, patients are treated with a regimen of 20 min of stimulation per session; such treatment regimens remain to be evaluated.

Unlike in vitro electrical stimulation studies, where electric fields and currents can be monitored precisely, in clinical settings, measuring the induced electric field and currents in wounds is technically challenging. Consequently, in most clinical studies, there is a lack of verification of directly measured electric fields and currents in the wounds on patients. So caution should be taken when interpreting clinical results.

Overall, electrical stimulation treatments are relatively safe. No major adverse events have been reported, although minor skin irritation is associated with continuous direct current or pulsed direct current between 50 and 1000 μA [48].

It is intriguing that at the single-cell level, the anode and cathode has different effects on cells—the polarity of the treatment electrode is important in managing chronic wounds in vivo. However, a recent review indicates that electrical stimulation improves wound blood flow and leads to wound size reduction in patients regardless of the polarity [42].

## Conclusions

Endogenous electric fields at wounds may play a major role in wound healing and regenerative processes, with possible mechanisms involving regulation of cell migration, cell differentiation, and tissue growth. Functional characterization of endogenous electric fields in mammalian development and wound healing has produced some significant insights. Major gaps in knowledge remain, on which electrical stimulation therapies ultimately depend. Electrical stimulation offers a unique treatment option to chronic and non-healing wounds. Greater understanding of the mechanisms will bring us closer to effective clinical applications. Additional experimental and clinical research are needed to elucidate how wound regeneration is affected by the selection of electric polarity and how this may affect the overall healing response. It is also imperative to determine the standardization of electrical stimulation across diverse devices, including the regimens and optimal parameters (polarity, current amplitude, frequency, and duration) in each clinical condition.
